# Effects of Thai native chicken breast meat consumption on serum uric acid level, biochemical parameters, and antioxidant activities in rats

**DOI:** 10.1038/s41598-022-18484-2

**Published:** 2022-08-18

**Authors:** Prapassorn Potue, Petcharat Chiangsaen, Putcharawipa Maneesai, Juthamas Khamseekaew, Poungrat Pakdeechote, Vibuntita Chankitisakul, Wuttigrai Boonkum, Natthaya Duanghaklang, Monchai Duangjinda

**Affiliations:** 1 Department of Physiology, Faculty of Medicine, Khon Kaen University, Khon Kaen, 40002 Thailand; 2 Faculty of Medicine, Bangkokthonburi University, Bangkok, 10170 Thailand; 3 Department of Animal Science, Faculty of Agriculture, Khon Kaen University, Khon Kaen, 40002 Thailand; 4 Network Center for Animal Breeding and Omics Research, Khon Kaen University, Khon Kaen, 40002 Thailand

**Keywords:** Nutritional supplements, Fat metabolism, Feeding behaviour, Metabolic diseases, Metabolomics

## Abstract

This study aimed to evaluate the effect of a high protein diet comprising breast meat from commercial broiler (BR), Thai native (PD), and commercial broiler × Thai native crossbred (KKU-ONE) chicken on serum uric acid, biochemical parameters, and antioxidant activities in rats. Male Sprague–Dawley rats were divided into four groups. The control group received a standard chow diet, and the other three groups were fed a high protein diet (70% standard diet + 30% BR, PD, or KKU-ONE chicken breast) for five weeks. The PD- and KKU-ONE-fed rats had lower plasma total cholesterol and triglyceride levels than the control rats. A decrease in HDL-c was also observed in rats fed a diet containing BR. Liver weight, liver enzyme, plasma ALP, xanthine oxidase activity, serum uric acid, creatinine, superoxide production, and plasma malondialdehyde levels increased in BR-fed rats. The findings of this study might provide evidence to support the use of Thai native and Thai native crossbred chicken breast meat as functional foods.

## Introduction

Chicken meat is high nutritional food due to its moderate energy content, highly digestible proteins, unsaturated lipids, B-group vitamins, and minerals. Chicken meat consumption is associated with a reduced risk of diet-related diseases, as well as cardiovascular diseases^1^. Chicken meat consumption continues to grow and remains universally popular among consumers. To meet the growing demand of consumers, fast-growing broilers are selected and raised in an evaporative cooling system, a poultry house with evaporative cooling pads for ventilating and cooling the house to suitable ambient temperature (15–25 °C). It has been reported that broiler meat has a higher fat content than native chicken meat^2,3^. The growth performance, feed efficiency, and meat yield of native chicken are inferior to those of broilers. However, they are very popular among native consumers because their meat quality and flavor are different from those of broilers ^4^. Chicken breast meat has recently been studied as a protein supplement for fitness and sports science for improving endurance^5^ and exercise performance^6^. Several studies have demonstrated that native chicken contains substantial amounts of bioactive compounds, such as angiotensin-converting enzyme (ACE) inhibitors^7^, anserine, and carnosine^8,9^. Therefore, native chicken meat has been promoted as a functional food with health benefits for consumers.

Thai native chicken, such as Pradu Hang Dam, have distinctive properties such as a unique flavor and a soft and firm texture^2^. Thai native chicken has greater muscle fiber diameter^10^, higher shear values^11^, and lower fat^12^ than broilers. The consumption of Thai native chicken, although relatively rare, is rapidly growing. A few years ago, the Network Center for Animal Breeding and Omics Research, Khon Kaen University, developed a commercial broiler × Thai native crossbreed named KKU-ONE, which contains 25% Thai native genetics. KKU-ONE breeding aims to improve meat quality and production costs to produce meat with lesser purine than commercial chicken meat to meet the demands of modern consumers. The new breed satisfies the ideal requirements of chicken meat as it is soft and tender; however, some of the special meat textures and tastes of the native breeds are conserved. Thai native crossbred chicken breasts have higher anserine and anserine/carnosine concentrations, as well as antioxidant properties, than commercial broiler chicken breasts^13^. In this regard, it might be useful as a functional meat source given its antioxidant dipeptides.

High protein diets are recommended and promoted to reduce body weight and gain lean body mass^14,15^. Chicken meat, particularly breast meat, is a source of high quality protein necessary for body function. However, broiler chicken breast meat has a higher fat content than native chicken breast meat^12^. A high level of fat causes diet-related diseases, including cardiovascular diseases. Moreover, chicken breast meat is recognized as a purine-rich food that increases uric acid, the main risk factor for inflammatory arthritis or gout^16,17^. Therefore, the safety profiles and effects of chicken breast meat consumption require further study. Chicken breast meat comprising bioactive compounds that prevents diet-related diseases or promotes health is an alternative choice for the modern consumers.

To promote native chicken breast meat as a functional food and establish them in modern trade, studies comparing the consumption of these chicken breeds are needed. However, there is no evidence regarding the effects of high protein consumption in various chicken breast meat in live animals. Therefore, this study aimed to compare the effects of a high protein diet comprising breast meat from three different chicken breeds, specifically examining serum uric acid levels, biochemical parameters, and antioxidant activities in rats.

## Results

### Amino acid composition and nutritive values of chicken breast meat

The amino acid profiles of BR, KKU-ONE, and PD breast meat are shown in Table 1. PD and KKU-ONE meat have higher contents of branched chain amino acids (leucine, isoleucine, and valine; BCAA), flavor related amino acids (valine, leucine, isoleucine, phenylalanine, proline), tasty- related amino acids (glutamic acid, alanine, glycine, serine, threonine), neurotransmitter- related amino acids (phenylalanine, tryptophan, tyrosine; NRAA), and total essential amino acids (histidine, isoleucine, leucine, lysine, methionine, threonine, tryptophan, and valine; EAA) than BR meat. Glycine, proline, and histidine levels were higher in PD than those in BR, whereas glutamic acid, lysine, and phenylalanine levels were higher in KKU-ONE than those in BR.

**Table 1:** The amino acid composition of breast meat from different chicken breeds.

	BR	KKU-ONE	PD
Alanine (mg/100 g)	1076	1370	1218
Aspartic acid (mg/100 g)	1108	1716	1466
Cystine (mg/100 g)	381	161	249
Glutamic acid (mg/100 g)	2792	4288	3457
Glycine (mg/100 g)	598	751	907
Histidine (mg/100 g)	1152	1290	1450
Hydroxylysine (mg/100 g)	< 20	< 20	< 20
Hydroxyproline (mg/100 g)	< 20	< 20	< 20
lsoleucine* (mg/100 g)	1461	1880	1791
Leucine* (mg/100 g)	2536	2888	3254
Lysine (mg/100 g)	4103	5411	3552
Methionine (mg/100 g)	278	517	587
Phenylalanine (mg/100 g)	1209	1719	1400
Proline (mg/100 g)	510	746	760
Serine (mg/100 g)	359	565	443
Threonine (mg/100 g)	483	726	553
Tryptophan (mg/100 g)	133	224	172
Tyrosine (mg/100 g)	1894	2017	2216
Valine* (mg/100 g)	1147	1561	1405
BCAA* (mg/100 g)	5144	6329	6450
Flavor-related AA (mg/100 g)	6863	8794	8610
Tasty-related AA (mg/100 g)	5308	7700	6578
Neurotransmitter-related AA (mg/100 g)	3586	3960	3788
Total essential AA (mg/100 g)	12,502	16,216	14,164

BR: Commercial broiler, KKU-ONE: commercial broiler × Thai native crossbred, PD: Thai native chicken (Pradu Hang Dam); BCAA: valine, leucine, isoleucine; Flavor-related AA: valine, leucine, isoleucine, phenylalanine, proline; Neurotransmitter-related AA: phenylalanine, tryptophan, tyrosine; Tasty-related AA: glutamic acid, alanine, glycine, serine, threonine; Total essential AA: histidine, isoleucine, leucine, lysine, methionine, threonine, tryptophan, valine.

The purine, anserine, and carnosine content in breast meat from different chicken breeds of BR, KKU-ONE, and PD breast meat are shown in Table 2. PD breast meat has the lowest contents of all purine bases. Xanthine and uric acid have not found in chicken breast meat of all breeds. PD and KKU-ONE breast meat have higher anserine and carnosine contents than BR.

**Table 2:** The purine, anserine, and carnosine content in breast meat from different chicken breeds.

	BR	KKU-ONE	PD
Purine (mg/100 g)	178.92 ± 5.19^a^	155.79 ± 3.41^b^	126.83 ± 4.51^c^
Adenine (mg/100 g)	39.32 ± 2.80^a^	33.00 ± 7.22^ab^	20.82 ± 5.95^b^
Guanine (mg/100 g)	56.59 ± 3.08^a^	42.06 ± 6.83^b^	39.58 ± 6.49^b^
Hypoxanthine (mg/100 g)	83.01 ± 0.80 ^a^	80.73 ± 2.28^a^	66.43 ± 0.95^b^
Xanthine (mg/100 g)	–	–	–
Uric acid (mg/100 g)	–	–	–
Anserine (mg/100 g)	556.50 ± 115.48^a^	1124.36 ± 58.02^b^	1098.35 ± 78.47^b^
Carnosine (mg/100 g)	133.58 ± 48.86	159.99 ± 10.21	174.18 ± 9.68

Data are expressed as the mean ± SEM. (n = 7 samples/group); BR: commercial broiler, KKU-ONE: commercial broiler × Thai native crossbred, PD: Thai native chicken (Pradu Hang Dam); Values within a row bearing different letters (a–c) are significantly different at *p* < 0.05.

The nutritive values in Table 3 reveal that both PD and KKU-ONE meat are leaner than BR meat. Moreover, KKU-ONE and PD meat have lower fat, saturated fat, and cholesterol content and higher protein content than BR meat. In addition, BR breast meat contains the highest sodium level. Interestingly, PD meat had more iron than other chicken breed meat.

**Table 3:** The nutritive values of breast meat from different chicken breeds.

	BR	KKU-ONE	PD
Energy (kcal/100 g)	376.97	377.19	377.19
Fat (g/100 g)	3.33	1.31	1.27
Saturated fat (g/100 g)	1.06	0.49	0.51
Cholesterol (mg/100 g)	312.47	238.97	243.03
Protein (g/100 g)	84.30	90.59	90.89
Carbohydrate (g/100 g)	2.45	0.76	0.55
Sodium (mg/100 g)	89.28	49.37	41.03
Calcium (mg/100 g)	43.91	44.70	45.53
Iron (mg/100 g)	1.96	1.89	4.17

BR: Commercial broiler, KKU-ONE: Commercial broiler × Thai native crossbred, PD: Thai native chicken (Pradu Hang Dam).

### Effect of high protein BR, KKU-ONE, or PD chicken breast meat diet on body and organ weight of rats

Over five weeks, there were no significant differences in the BW of different group rats. However, visceral and epididymal fat contents in KKU-ONE or PD group rats significantly decreased than that in the control group rats (*p* < 0.05). In addition, BR, KKU*-*ONE, and PD group rats showed a significant increase in liver and kidney weight from control group rats (*p* < 0.05; Table 4).

**Table 4:** Effect of a high protein diet comprising breast meat from different chicken breeds on body and organ weight of rats.

Parameter	CON	BR	KKU-ONE	PD
BW (g)	503.00 ± 12.99	482.70 ± 6.48	483.20 ± 5.23	482.10 ± 5.19
Visceral fat content (g)	9.20 ± 0.92	6.93 ± 0.49	6.04 ± 0.56*	6.19 ± 0.41*
Visceral fat content/BW (mg/g)	18.20 ± 1.36	14.29 ± 0.96	11.35 ± 0.93*	12.92 ± 0.88*
Epididymal fat content (g)	7.48 ± 0.47	6.18 ± 0.29*	5.21 ± 0.23*	5.23 ± 0.25*
Epididymal fat content/BW (mg/g)	14.87 ± 0.89	12.67 ± 0.56	11.23 ± 0.28*	10.85 ± 0.48*
Kidney weight (g)	3.46 ± 0.09	3.82 ± 0.05*	3.67 ± 0.07	3.78 ± 0.09*
Kidney weight/BW (mg/g)	6.90 ± 0.22	7.87 ± 0.10*	7.51 ± 0.09*	7.82 ± 0.18*
Liver weight (g)	13.39 ± 0.35	15.20 ± 0.26*	14.88 ± 0.44*	15.00 ± 0.37*
Liver weight/BW (mg/g)	27.26 ± 0.57	31.16 ± 0.61*	30.82 ± 0.78*	30.96 ± 0.74*

Data are expressed as the mean ± SEM. (n = 8 animals/group); * *p* < 0.05 vs. CON; BW: body weight; BR: commercial broiler group, CON: control group, KKU-ONE: commercial broiler × Thai native crossbred group, PD: Thai native chicken (Pradu Hang Dam) group.

### Effect of high protein BR, KKU-ONE, or PD chicken breast meat diet on plasma lipid profiles in rats

**Figure 1:**
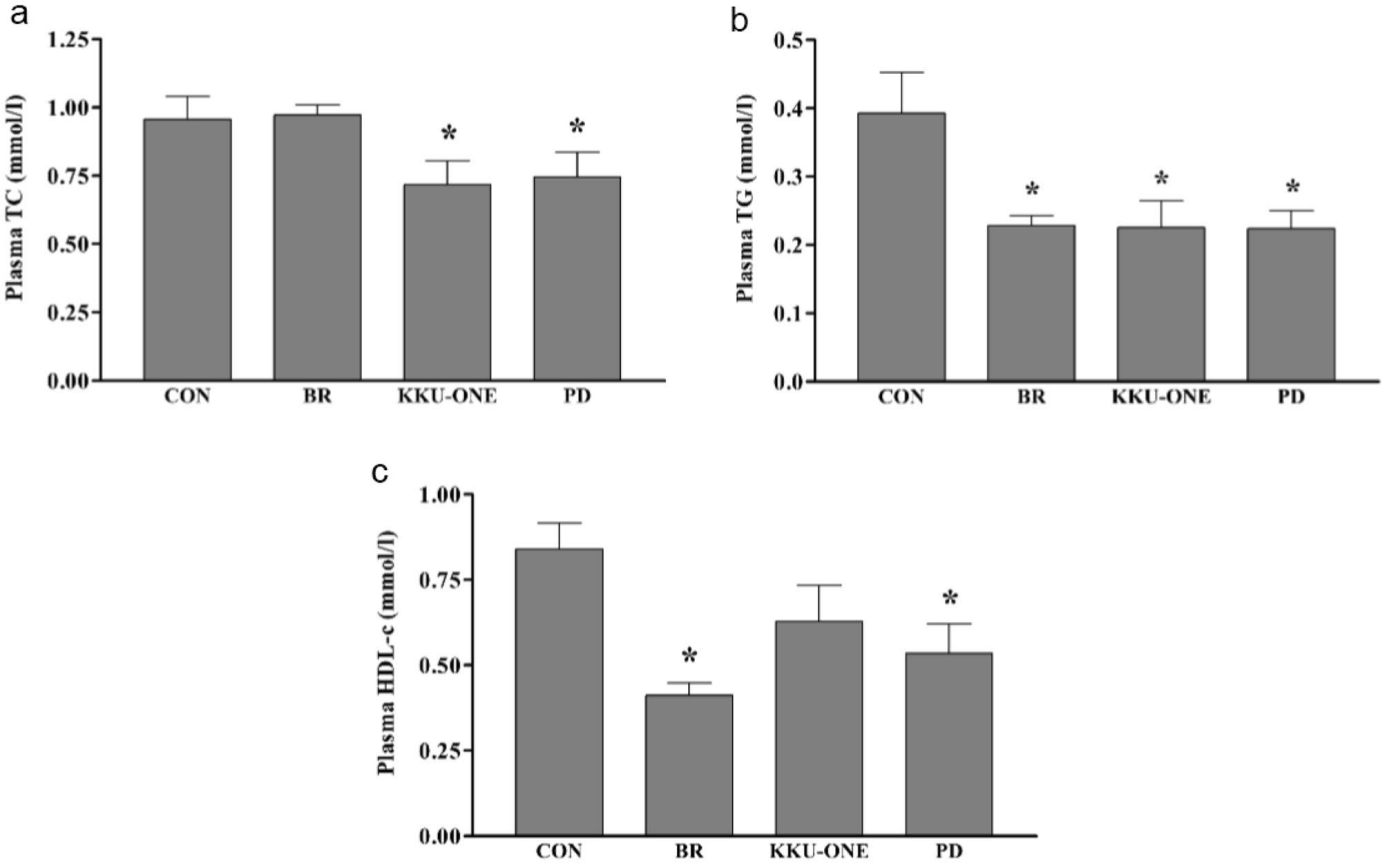
Effect of high protein chicken breast meat diet on plasma (**a**) TC, (**b**) TG, and (**c**) HDL-c in rats. Data are expressed as means ± SEM. (n = 6–8 animals/group); *p < *0.05* vs. CON; TC: total cholesterol, TG: total triglyceride, HDL-c: high-density lipoprotein cholesterol; BR, commercial broiler group, CON, control group, KKU-ONE, commercial broiler × Thai native crossbred group, PD, Thai native chicken (Pradu Hang Dam) group.

Five weeks of the high protein diet comprising KKU-ONE and PD chicken breast meat reduced plasma cholesterol and triglycerides than the standard chow diet (*p* < 0.05; Fig. 1a and b). In addition, the plasma HDL-c concentration in KKU-ONE group rats was similar to that in the control group rats, whereas HDL-c concentration in other group rats was lower than that in the control group rats (Fig. 1c).

### Effect of high protein BR, KKU-ONE, or PD chicken breast meat diet on xanthine oxidase activity and uric acid levels in rats

**Figure 2:**
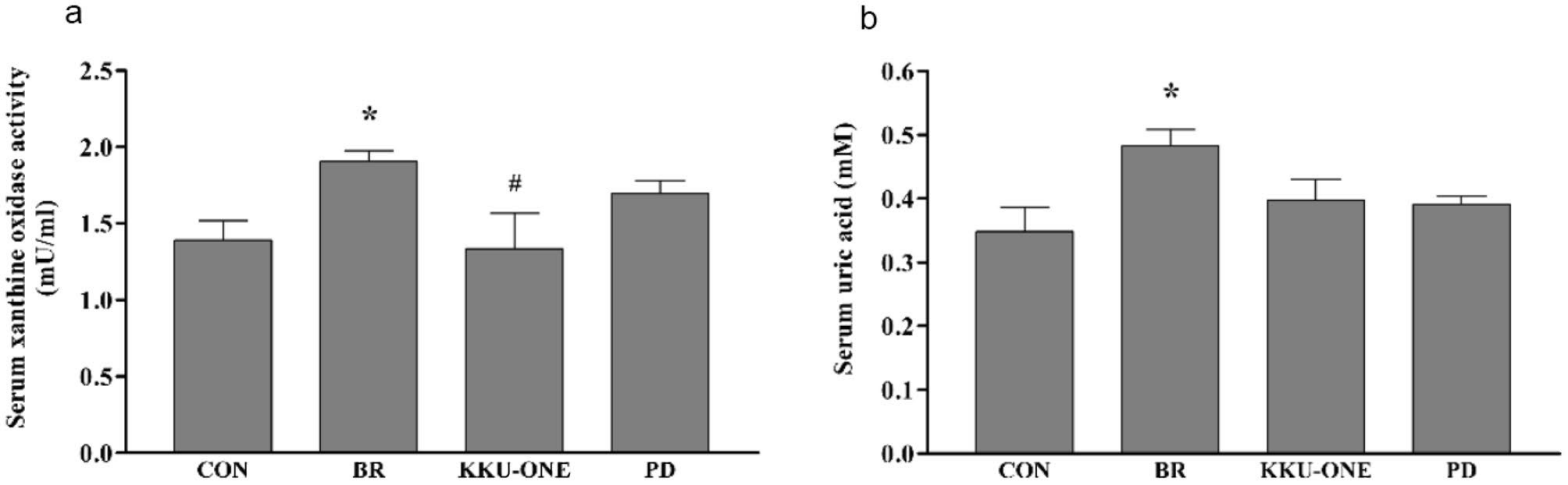
Effect of high protein chicken breast meat diet on (**a**) serum xanthine oxidase activity and (**b**) uric acid levels in rats. Data are expressed as means ± SEM. (n = 5–7 animals/group); *p < 0.05 vs. CON, #*p* < 0.05 vs. BR; CON: control group, BR: commercial broiler group, KKU-ONE: commercial broiler × Thai native crossbred group, PD: Thai native chicken (Pradu Hang Dam) group.

Serum xanthine oxidase activity and uric acid levels in the BR group rats were significantly higher than those in the control group rats (*p* < 0.05). Interestingly, serum xanthine oxidase activity in the BR group rats was significantly higher than that in the KKU-ONE group rats (*p* < 0.05; Fig. 2).

### Effect of high protein BR, KKU-ONE, or PD chicken breast meat diet on renal function in rats

**Figure 3:**
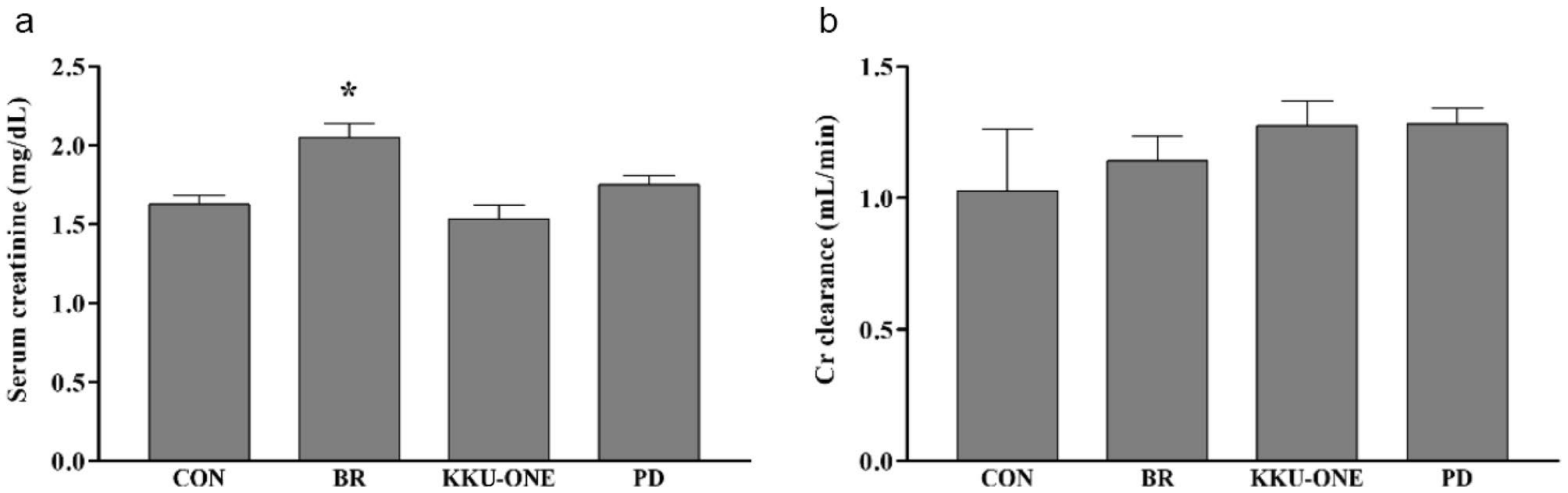
Effect of high protein chicken breast meat diet on (**a**) serum creatinine levels and (**b**) creatinine clearance in rats. Data are expressed as means ± SEM. (n = 5–7 animals/group); *p < 0.05 vs. CON. Cr: creatinine; BR: commercial broiler group, CON: control group, KKU-ONE: commercial broiler × Thai native crossbred group, PD: Thai native chicken (Pradu Hang Dam) group.

Serum creatinine levels in the BR group rats were significantly higher than those in the control group rats (*p* < 0.05; Fig. 3a). However, there was no significant difference in the creatinine clearance rates between the groups (Fig. 3b).

### Effect of high protein BR, KKU-ONE, or PD chicken breast meat diet on serum ACE activity in rats

**Figure 4:**
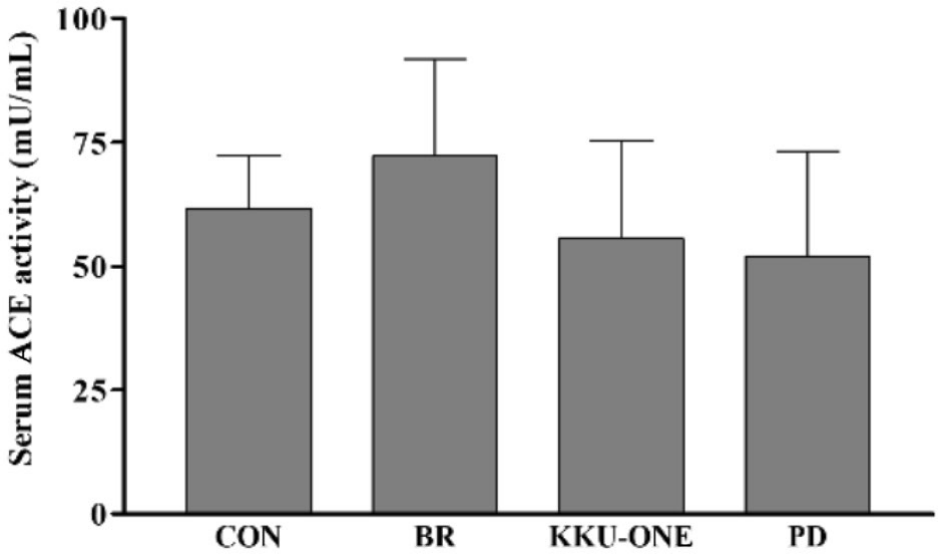
Effect of high protein chicken breast meat diet on serum ACE activity in rats. Data are expressed as means ± SEM. (n = 5–6 animals/group); *p < 0.05 vs. CON; ACE: angiotensin converting enzyme; BR: commercial broiler group, CON: control group, KKU-ONE: commercial broiler × Thai native crossbred group, PD: Thai native chicken (Pradu Hang Dam) group.

Serum ACE activity was not significantly different among all groups (Fig. 4).

### Effect of high protein BR, KKU-ONE, or PD chicken breast meat diet on oxidative stress markers in rats

**Figure 5:**
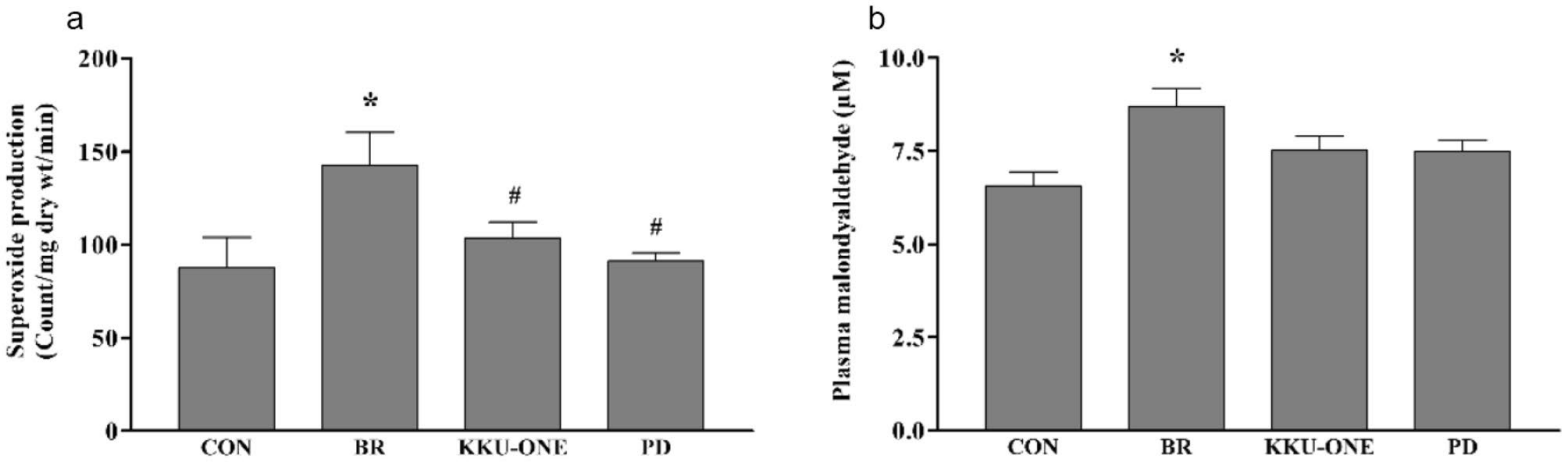
Effect of high protein chicken breast meat diet on (**a**) vascular O2•-production and (**b**) plasma MDA levels in rats. Data are expressed as means ± SEM. (n = 6–8 animals/group); *p < 0.05 vs. CON, #*p* < 0.05 vs. BR; MDA: malondialdehyde; BR: commercial broiler group, CON: control group, KKU-ONE: commercial broiler × Thai native crossbred group, PD: Thai native chicken (Pradu Hang Dam) group.

O2•- production in carotid arteries, as well as the end-product of lipid peroxidation and plasma MDA, increased in BR group rats than those in the control group rats (*p* < 0.05). KKU-ONE and PD group rats showed significantly decreased vascular O2•- production than the BR group rats (*p* < 0.05; Fig. 5).

### Effect of a high-protein diet of BR, KKU-ONE and PD chicken meat on plasma liver enzymes in rats

**Figure 6:**
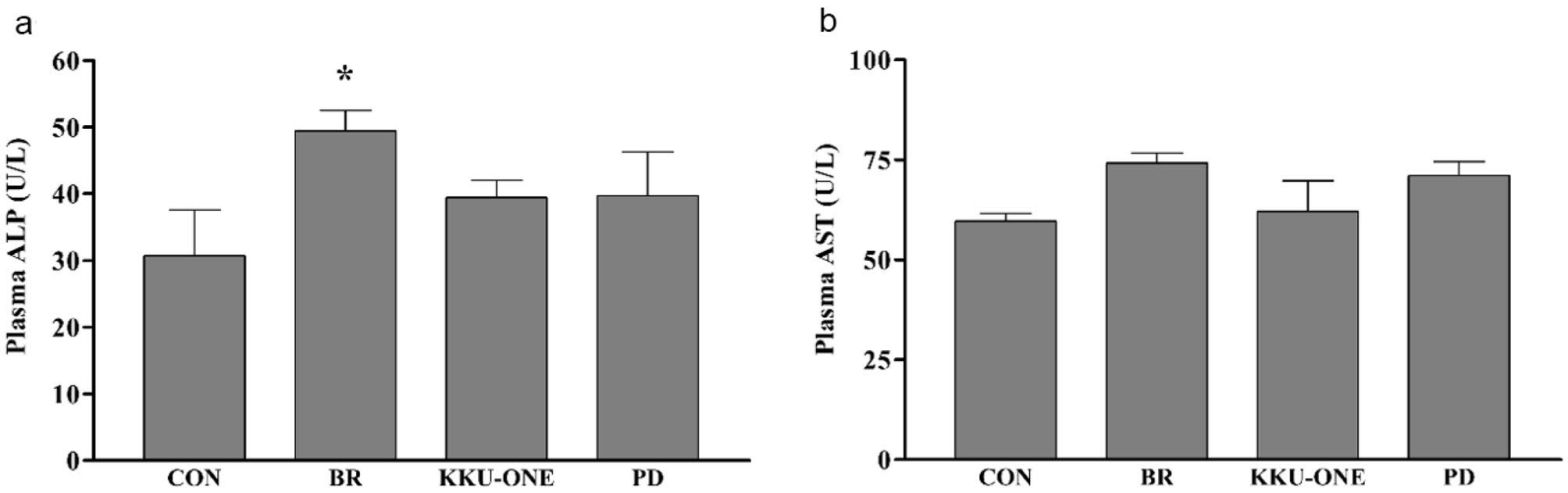
Effect of high-protein diet from chicken meat on (**a**) plasma ALP and (**b**) plasma AST in rats. Data are expressed as means ± SEM. (n = 7 animals/group). *p < 0.05 vs. CON. ALP: alkaline phosphatase, AST: aspartate transaminase, CON: control group, BR: commercial broiler group, KKU-ONE: Thai native crossbred chicken group, PD: Pradu Hang Dam (Thai native chicken) group.

Liver enzymes, including ALP and AST, were measured to determine liver function. The results showed that rats fed with a high-protein diet in the BR group had high plasma ALP levels compared with the control group (*p* < 0.05) (Fig. 6a). There were no differences between groups in terms of plasma AST levels (Fig. 6b).

## Discussion

The higher contents of BCAAs and EAA in PD and KKU-ONE breast meat compared to BR revealed the benefits for muscle synthesis as reported in^18^. The level of glutamic acid, the most important amino acid for enhancing the flavor of meat, was higher in PD and KKU-ONE meat than that in BR meat, which is in agreement with the results of a previous study^8^. Subsequently, fewer flavor-related amino acids and tasty-related amino acids were found in BR than those in PD and KKU-ONE. The benefits in flavor and taste in native chicken compared to those in broilers have been supported by several reports^19,20^. The higher anserine and lower purine contents in PD and KKU-ONE breast meat may improve of exercise performance with low-risk of high serum uric acid. This study demonstrated that a high protein diet from three different chicken breeds BR, KKU-ONE, and PD reduced fat content and plasma lipid profiles in rats. BR group rats showed low HDL-c levels and high plasma ALP, xanthine oxidase activity, serum uric acid, serum creatinine, and oxidative stress marker levels. The significant reductions of HDL-c concentration in rats received PD in the present study is consistent with the reductions of TC and TG in the PD rats. It might not be relevant to HDL-c synthesis. Moreover, PD chicken has lower contents of fat and carbohydrate and that could reduce lipid sources and synthesis^21,22^. However, the high protein diet comprising KKU-ONE and PD chicken breast meat did not alter biochemical parameters or oxidative stress markers. Interestingly, the KKU-ONE group rats showed lower xanthine oxidase activity and O2•- production than the BR group rats. This difference might be due to the different bioactive compounds and antioxidant properties in each chicken breed.

Chicken breast meat is a nutritionally valuable food with a lower fat content than other types of meat^23^. This study also showed that PD and KKU-ONE breast meat had lower fat and cholesterol levels than BR breast meat. Dietary protein from chicken meat is widely accepted for weight loss and obesity prevention^24,25^. However, these effects were not observed in this study. Navas-Carretero et al.^26^ have demonstrated that the frequent consumption of moderately high protein from chicken meat reduces weight and fat mass in adults. The higher BCAA, alanine, and glutamic acid contents in PD and KKU-ONE meat than those in BR meat might provide slight benefits for muscle synthesis. This study suggests that a high protein diet using chicken breast meat, with a decreased carbohydrate proportion, reduces fat content and plasma lipid profiles in PD- and KKU-ONE group rats than that in the control group rats. A high protein and low-carbohydrate diet reduces weight and fat mass and is involved in changes in mitochondrial oxidation and energy expenditure^27^. Moreover, protein intake is more satiating than carbohydrate or fat intake at identical energy values^28^. However, fat content and total cholesterol in the BR group rats were similar to those in the control group rats, and tended to be higher than those in the PD- and KKU-ONE group rats. This may be due to the high fat content in broiler meat^28^. Globally, commercial breeding companies are focusing on chicken growth performance using fast-growing broiler strains with intensive fattening systems. Although the fatty acid profile of muscle lipids is mostly affected by feeding, genotype also affects fatty acids related to human health^29^. Native chicken breast meat seems to be beneficial in terms of human health because of its low fat content and fatty acid profiles^4^. A recent study has reported that fat deposition in Thai native chicken is also related to Peroxisome Proliferator-Activated Receptor (PPAR)^30^ and adipocyte type fatty acid binding protein (A-FAB) gene expression^12^.

The liver is one of the organs affected by adaptation to high protein intake, increasing liver enzyme activity^31,32^. Chronic high protein intake increases liver weight/BW ratio and induces hepatic injury/inflammation. This mechanism is involved in amino acid metabolism in the liver^32^. In this study, we found that liver weight increased in all high protein diet-fed rats. Moreover, ALP level in plasma increased after consuming a high protein diet comprising BR chicken breast meat, while it was within normal levels after consuming a high protein diet comprising PD or KKU-ONE chicken breast meat. However, plasma AST levels did not change in any of the groups. The amount of protein content and experimental period of this study might not be high enough to produce detrimental effects on liver function. However, in the longer periods of time with higher protein content, it might be harmful effects such as hepatic injury and inflammation in rats^33^. In addition, there is no scientific evidence confirmed the detrimental effects of long-term high protein diet on liver function in healthy humans^34^.

A high intake of purine-rich foods such as beef, seafood, and chicken is associated with high serum uric acid levels and increased gout risk^16,35^. Uric acid is the end-product of purine nucleotide metabolism. Purine bases are converted to hypoxanthine and xanthine by many enzymes. Then, hypoxanthine is oxidized to xanthine and subsequently to uric acid by xanthine oxidase^36^. Chicken contains high amount of total purines with a high ratio of hypoxanthine, which can be an increased risk factor for developing gout^17^. High levels of serum uric acid were observed in BR group rats in this study. High protein intake from PD and KKU-ONE did not change the serum uric acid level related to xanthine oxidase activity. Due to the breeding goal of native chicken improvement, the KKU-ONE chicken breed was developed for both improved meat quality and decreased purine content. The results of this study indicate that KKU-ONE chicken breast meat contains less purines than commercial broiler meat. However, further investigations are needed to confirm the amount of purine and/or purine bases in each chicken breed.

It has been hypothesized that high protein intake may promote renal damage by chronically increasing glomerular pressure and hyperfiltration^37^. The effect of increased protein intake on renal alterations related to renal hypertrophy has been demonstrated in mice^38^. In this study, an increase in kidney weight and relative kidney weight was observed in rats fed a high protein diet comprising chicken meat. In addition, this study demonstrated an association between serum uric acid level and kidney function. Uricase, the enzyme that degrades uric acid, in knockout mice showed hyperuricemia, with elevated serum creatinine and blood urea nitrogen, indicating that uric acid is related to renal dysfunction^39^. Consistent with the results of this study, serum uric acid and creatinine levels were high in BR group rats. Serum creatinine of BR group rats was higher than that of the control group rats, and there were no differences between that in the control and PD or KKU-ONE group rats. This might be due to changes in muscle mass, dietary protein intake, or creatine supplementation^40^, as well as creatine and creatinine content in chicken breast meat^41^. Dietary protein can contribute to increased creatine and creatinine intake, thus increasing serum creatinine levels^42,43^. Serum and urine creatinine concentration is commonly used for determining renal function as an estimation of GFR, as a high protein diet increases GFR by glomerular hyperfiltration^44^. However, a high protein diet comprising chicken meat did not affect kidney function, since there was no difference in the GFR between all groups in this study. Lacroix et al.^45^ reported no abnormalities in renal function or pathology in male rats on a long-term high protein diet. Although excessive protein intake remains a health concern because of its effect on renal alterations, changes in renal function are likely a normal adaptive mechanism for a high protein diet within the functional limits of healthy kidneys^37^.

In addition to their nutritional properties, proteins from various types of meat provide ACE-inhibitory peptides released through digestion processes in the gastrointestinal tract by appropriate enzymes^46^. ACE is the key enzyme in the renin–angiotensin–aldosterone system, which plays critical roles in maintaining physiological homeostasis. ACE converts inactive angiotensin I into angiotensin II to regulate many physiological and pathological responses. ACE inhibition is well known, as many common drugs use this interaction to treat hypertension^47^. Several studies have revealed the presence of ACE-inhibitory peptides in chicken breast meat^7,48^. The bioavailability of ACE inhibitory peptides is determined by their resistance to peptidase degradation and intestinal absorption, which can either activate or inactivate bioactive peptides^49^. Moreover, the ACE-inhibitory activity of peptides in chicken breast meat depends on the time and temperature at which meat is cooked^7^. In this study, high protein intake from different chicken breeds did not alter ACE activity compared to that from normal diets. This might be due to the variation in ACE inhibitory activity caused by the processes of cooked meat, as well as the bioavailability of peptides in digestion and absorption processes. In addition, muscle composition of the different breeds might affect protein structure and peptides, which could lead to varied ACE inhibitory activity.

Several studies have reported the antioxidant effects of chicken breast meat containing bioactive compounds^8,50^. Anserine and carnosine dipeptides found in chicken breast meat possess strong and specific antioxidant properties. A high percentage of total carnosine-related compounds, including carnosine, anserine, and homocarnosine, and low TBARS, are found in chicken breast, indicating low lipid oxidation values^51^. In addition, the antioxidant properties of anserine and carnosine in chicken have been reported previously^8,52,53^, and we found that PD and KKU-ONE group rats had lower vascular O2•- production than BR group rats, which did not change the level of plasma MDA, a lipid peroxidation marker. In addition, high protein consumption from BR increases both vascular O2•- production and plasma MDA. This finding confirmed the antioxidant properties of a high protein diet from Thai native and Thai native crossbred chicken, which is in agreement with a previous report^29^ that the anserine, anserine/carnosine, and antioxidant activity of the CH breed and its crossbred KKU-ONE were higher than those in the commercial broiler chicken breed.

## Conclusions

This study demonstrated the changes in biochemical parameters after consuming a high protein diet comprising Thai native and Thai native crossbred chicken from that after consuming a high protein diet comprising commercial broiler chicken. The results showed that a high protein broiler chicken breast meat diet decreased lipid profiles but increased liver enzyme, uric acid, creatinine concentration, and oxidative stress markers, which might be harmful to health. However, the high protein Thai native and Thai native crossbred chicken breast meat diet had a lipid-lowering effect by decreasing fat content and lipid profiles. Moreover, the other biochemical parameters and oxidative stress did not change in rats consuming Thai native and Thai native crossbred chicken breast meat. Therefore, Thai native and Thai native crossbred chicken may have beneficial effects as functional foods compared to commercial broilers.

## Methods

### Animal and study design

Six-week-old male Sprague–Dawley rats (220–260 g) were used in this study. The animals were purchased from Nomura Siam International Co., Ltd. (Bangkok, Thailand) and housed at a controlled temperature (23 ± 2 °C) with a 12 h dark–light cycle and 30–60% relative humidity under pathogen-free conditions. All animal procedures were completed in accordance with the ethical guidelines for the Care and Use of Laboratory Animals, which was reviewed and approved by the Animal Ethics Committee of Khon Kaen University (IACUC-KKU-63/63), based on the ethics for animal experimentation of the National Research Council of Thailand. Commercial broiler (BR) breast meat was purchased from a commercial store in Khon Kaen, Thailand. Thai native crossbred chicken (KKU-ONE; 25% Thai native) and Thai native chicken (Pradu Hang Dam, PD; 100% Thai native) breast meat were obtained from the Network Center for Animal Breeding and Omics Research, Khon Kaen University, Thailand.

After one week of acclimatization, the animals were randomly divided into four groups with eight animals in each group. Rats were housed in cage size 37.5 × 48 × 21 cm, with 4 rats/cage. Four groups of diets were fed. Group 1: Control group (standard chow diet); Group 2: BR group (70% standard chow diet + 30% BR breast meat); Group 3: KKU-ONE group (70% standard chow diet + 30% KKU-ONE breast meat); Group 4: PD group (70% standard chow diet + 30% PD breast meat). The chemical composition and gross energy (GE) of all diets used in this study was determined by the Animal Nutrition Laboratory, Department of Animal Science, Faculty of Agriculture, Khon Kaen University (Table 5). Metabolizable energy (ME) from chemical composition was calculated based on the formula suggested in ^54^: ME (KJ/g) = 18.4*CPdigest + 39.4*EEdigest + 15.2*CFdigest + 17.5*NFEdigest, where the digestibility (digest) for chemical compositions for rats was obtained from^55^. The control or standard diet contained 25.41% crude protein (CP), while the standard diet mixed with BR, KKU-ONE, or PD breast meat contained approximately 41.52–43.79% CP. Diet containing 30%^56^, 35%^57^, and 55%^58^ CP are considered high protein diets. Therefore, BR, KKU-ONE, and PD chicken breast meat, which contained more than 40% CP, could be considered a high protein diet. The GE for all diets were in a range of 17.05–18.12 kJ/g (4.08–4.30 KCal/g), and ME were in a range of 14.84–15.05 kJ/g (3.55–3.60 KCal/g). National Research Council suggest a diet containing GE of 17–19 kJ/g (4.0–4.5 KCal/g) and ME at least 15.0 kJ/g (3.6 KCal/g) will meet the energy requirement for maintenance and growth in rats^59^.

**Table 5:** Chemical composition of diets comprising breast meat from different chicken breeds.

Diet composition	CON	BR	KKU-ONE	PD
Carbohydrate, NFE (%)	57.84	41.57	41.03	41.43
Crude protein, CP (%)	25.41	41.52	43.61	43.79
Crude fat, EE (%)	1.79	2.87	1.92	1.63
Crude fiber, CF (%)	1.36	0.92	0.46	0.24
Moisture (%)	7.42	7.63	7.7	7.64
Ash (%)	6.18	5.5	5.28	5.26
GE (KJ/g)	17.05	18.12	17.56	17.93
ME (KJ/g)	14.87	15.05	14.90	14.84

CON, control; BR, commercial broiler; KKU-ONE, commercial broiler × Thai native crossbred; PD, Thai native chicken (Pradu Hang Dam); NFE = Nitrogen free extract; CP = Crude protein; EE = Ether extract; GE = Gross energy; ME = Metabolizable energy.

All diets for rats were prepared in pellet form. Chicken breasts were cooked and mixed with the standard diet. As fat is not uniformly distributed in chicken breast, entire ground skinless breasts were used instead of chopping them into small pieces to avoid obtaining large lean or fat portions. Then, blended and mixed with the standard chow diet at the ratio, standard chow diet : chicken meat, 70: 30. Each rat received diet pellets for 30 g/day in average. Distilled water was provided to the rats ad libitum.

The experiment was performed for five weeks. The body weight (BW) of all rats were measured weekly throughout the study. At the end of the study, the rats were anesthetized by peritoneally injecting 70 mg/kg thiopental sodium and euthanized using exsanguination. Blood samples were obtained from the abdominal aorta for the biochemical assays. Tissue samples, including liver, kidney, epididymal fat pads, and visceral fat pads were collected by dissection. All tissues were washed with saline solution and weighed. Data are expressed as tissue weight (g) and the relationship between regional tissue weights (mg)/BW (g).

### Amino acid composition; purine, anserine, and carnosine contents; and nutritive values of chicken breast meat

Amino acid profiles for each chicken breast meat type were analyzed using GC–MS according to the Association of Official Analytical Chemists (AOAC) Official Method 2002 No. 994.12 & 998.15^60^. The nutritive values of chicken breast meat were analyzed using the method of analysis for nutrition labeling AOAC International at Central Laboratory (Thailand) Co. Ltd.

Chicken breast meat from each breed were randomly sampling for seven replications to analyze total purine, anserine and carnosine contents. Total purine content was calculated from the combined amounts of adenine, guanine, hypoxanthine, and xanthine as described in^17^. The purine bases were separated on an Asahipak GS-320 HQ column (7.5 × 300 mm, 6-μm particles; Showa Denko America, Inc.; New York, NY, US) equipped with a High Performance Liquid Chromatography (HPLC) system (HP1260; Agilent Technologies; Santa Clara, CA, US). The mobile phase consisted of 150 mM sodium phosphate monobasic (NaH_2_PO_4_) at pH 2.5. The flow rate was 1.0 mL/min and the bases were detected by their absorbance at 260 nm. The adenine, guanine, hypoxanthine, xanthine, and uric acid quantities were determined by comparing the peak area with that of the external standards obtained from Sigma-Aldrich Co. (St. Louis, MO, USA).

Anserine and carnosine were separated on an Atlantis HILIC silica column (4.6 × 150 mm, 2.7 μm; Millipore) equipped with an HPLC system (HP1260; Agilent Technologies). The mobile phase A contained 0.65 mM ammonium acetate in acetonitrile (25:75 ratio) at a pH of 5.5; the mobile phase B contained 0.65 mM ammonium acetate in acetonitrile (70:30 ratio) at a pH of 5.5. A linear gradient from 100% phase A to 100% phase B in 25 min with 0.8 mL/min flow rate. The compounds were detected at 214 nm. The content of compounds was calculated using the standard curve for anserine and carnosine standards obtained from Sigma-Aldrich Co.

### Biochemical parameters

Total cholesterol (TC), triglyceride (TG), and high-density lipoprotein cholesterol (HDL-c) concentrations were determined using specific commercial kits (Human Gesellschaft fuer Biochemica und Diagnostica mbH; Wiesbaden, Germany). Aspartate transaminase (AST) and alkaline phosphatase (ALP) levels were measured by the Clinical Chemistry Laboratory Unit of the Faculty of Associated Medical Sciences, Khon Kaen University, Thailand. Serum xanthine oxidase activity was determined using the Xanthine Oxidase Activity Assay Kit (ab102522, Abcam Plc; Cambridge, UK). Serum uric acid levels were determined using the Uric Acid Assay Kit (ab65344, Abcam Plc).

### Renal function measurement

Urine samples were collected at the fifth week of the experiment by housing the rats in metabolic cages (Harvard Apparatus; Holliston, MA, USA) for 24 h. Urine volumes were recorded and blood samples were collected from the lateral tail vein. Urine and blood samples were centrifuged at 3500 rpm for 15 min and immediately stored at − 80 °C. Serum and urine creatinine levels were measured using a creatinine assay kit (Sigma-Aldrich). The creatinine clearance rate (Ccr) was used to estimate the glomerular filtration rate (GFR) and calculated using the standard formula: urinary flow rate × urine creatinine level/serum creatinine level.

### Assay of ACE activity and oxidative stress markers

The O-phthalaldehyde-chromogenic reaction was used to measure serum ACE activity, as previously described^61^. Carotid arteries were collected to determine O2•- production levels using lucigenin-enhanced chemiluminescence and the plasma MDA levels were detected using thiobarbituric acid reactive substances (TBARS) as previously described^62^.

### Statistical analysis

The results are expressed as the mean ± SEM. Data were analyzed using one-way analysis of variance (ANOVA) followed by Tukey’s post hoc test. Differences were considered statistically significant at *p*-value less than 0.05.

### Ethical approval

Animal ethics approval was granted by the Animal Ethics Committee of Khon Kaen University. All methods were carried out in accordance with relevant guidelines and regulations. This study is reported in accordance with ARRIVE guidelines.

## Data Availability

The datasets used and analyzed during the current study available from the corresponding author on reasonable request.
